# A Case Report on Spontaneous Coronary Artery Dissection and Its Potential Correlation With Fibromuscular Dysplasia

**DOI:** 10.7759/cureus.69017

**Published:** 2024-09-09

**Authors:** Franshesca E Gonzalez, Emily Dickinson, Guillermo Izquierdo-Pretel, Cesar E Mendoza

**Affiliations:** 1 Internal Medicine, Florida International University, Herbert Wertheim College of Medicine, Miami, USA; 2 Cardiovascular Disease, Jackson Memorial Hospital, Miami, USA

**Keywords:** fibromuscular dysplasia, acute coronary syndrome, acute chest pain, median arcuate ligament syndrome, spontaneous coronary artery dissection requiring intervention

## Abstract

This is a presentation of a common symptom, acute chest pain, with a rare etiology and the relevant implications of spontaneous coronary artery dissection (SCAD) and fibromuscular dysplasia (FMD). In a tertiary hospital in South Florida, during the fall of 2023, an adult female patient with acute chest pain was admitted to the internal medicine ward. The ECG demonstrated no acute ischemic changes. Troponin I levels were initially low upon admission at 0.012 ng/mL, borderline at four hours at 0.087 ng/mL, and increased to 9.49 ng/mL after eight hours. The patient was immediately taken for catheterization due to concerns of a high-risk condition, which revealed two SCADs: a mid-left anterior descending artery type 3 and a mid-posterior left ventricular artery type 2. Computed tomography angiography of the abdomen and pelvis demonstrated hooked morphology of the celiac trunk, with evidence of increased peak velocity of the celiac artery, typically seen in median arcuate ligament syndrome. However, the patient presented no symptoms such as abdominal pain, nausea, or vomiting. FMD was considered a clinical diagnosis as it explains the SCAD in the absence of other risk factors. In patients with coronary artery dissection and no other risk factors, it is crucial to consider the likelihood of FMD as an underlying cause and to evaluate the patient for different manifestations of FMD.

## Introduction

Spontaneous coronary artery dissection (SCAD) is a condition resulting in myocardial ischemia caused by a tear in the wall of the coronary artery. It is a rare cause of chest pain and is not associated with risk factors such as iatrogenic, traumatic, or atherosclerotic conditions. It is more prevalent in females younger than 60 years old and is one of the leading causes of acute coronary syndrome among females without risk factors such as hypertension, diabetes, hyperlipidemia, smoking, or obesity [1]. The diagnosis of SCAD is made by coronary angiography, and treatment involves either conservative management or percutaneous intervention depending on the severity. A common finding among patients with SCAD is evidence of hormonal effects, shear stress, or vascular involvement, like fibromuscular dysplasia (FMD). The gold standard for diagnosing FMD is conventional catheter-based angiography. We present a case of a patient with atypical chest pain who was found to have SCAD, reported here to discuss the concomitant relation with FMD.

## Case presentation

A 59-year-old female with a past medical history of patent foramen ovale, essential hypertension, mild hypercholesterolemia, chronic bilateral mild to moderate knee pain, and chronic hot flashes presented to a tertiary hospital after an episode of tight, burning anterior chest pain. The severe pain lasted 45 minutes and was associated with shortness of breath and sweating. She reported that the pain started while she was at rest during work and worsened when she attempted to exert herself. The patient had never experienced symptoms like this in the past and reports never having chest pain prior to this episode. Vitals obtained in the emergency department were within the normal range, except for an elevated systolic blood pressure of 141 mmHg (normal <120 mmHg). The physical exam was unremarkable, except for the patient appearing mildly anxious. The chest pain, shortness of breath, and diaphoresis had resolved. Labs on admission were significant for elevated white blood cell count, cholesterol, triglycerides, glucose, and rheumatoid factor (Table 1). The troponin value on admission was 0.012 ng/mL. At hours 4, 6, 8, 12, 27, and 62, the troponin values were 0.872 ng/mL, 3.15 ng/mL, 9.49 ng/mL, 3.350 ng/mL, and 1.850 ng/mL (normal < 0.035 ng/mL). 

**Table 1:** Laboratory results at the time of admission. LDL: Low-density lipoprotein; HDL: High-density lipoprotein.

Laboratory test	Patient values	Reference values
Complete Blood Count		
WBCs (k/mm^3^)	11.8	4.0-10.5
Neutrophils %	47.2	36.0-70.0
Lymphocytes %	46	16.0-43
Monocytes	4.0	6.0-12.0
Hemoglobin (g/dL)	13.3	11.1-14.6
Hematocrit (%)	41.0	33.2-43.4
Platelets (x103/mcl)	272	140-400
Complete Metabolic Panel		
Sodium (mmol/L)	139	137-145
Potassium (mmol/L)	3.7	3.6-5
Chloride (mmol/L)	98	98-107
Carbon dioxide (mmol/L)	27	22-30
Blood urea nitrogen (mg/dL)	10	7-17
Creatinine (mg/dL)	0.60	0.52-1.04
Glucose (mg/dL)	116	74-106
Calcium (mg/dL)	9.9	8.4-10.2
Total bilirubin (mg/dL)	0.5	0.2-1.3
Total protein (g/dL)	7.3	6.3-8.2
Albumin (g/dL)	4.8	3.9-5.0
Alkaline phosphatase (U/L)	74	38-126
Aspartate aminotransferase (U/L)	29	15-46
Alanine aminotransferase (U/L)	28	9-52
Lipase (U/L)	113	23-300
C-reactive protein (mg/L)	<0.5	0.0-0.9
Troponin I Trend* (ng/mL)	0.012→ 0.872→ 3.15→ 9.49 →3.350→ 1.850	0.035-0.119 = Borderline Results >0.119: Highly indicative of an "MI"
Erythrocyte sedimentation (mm/hr)	10	0-20
Cholesterol (mg/dL)	222	150-200
Calculated LDL (mg/dL)	125	65-160
HDL (mg/dL)	67	40-60
Triglycerides (mg/dL)	152	35-135
Thyroid-stimulating hormone (mcIU/mL)	1.27	0.270-4.200
Free T4 (ng/dL)	0.97	0.8-1.8
Rheumatoid Factor (IU/ml)	23	0-13
*Troponin I values were recorded at the time of admission, followed by measurements at intervals of 4, 6, 8, 12, 27, and 62 hours.		

**Figure 1:**
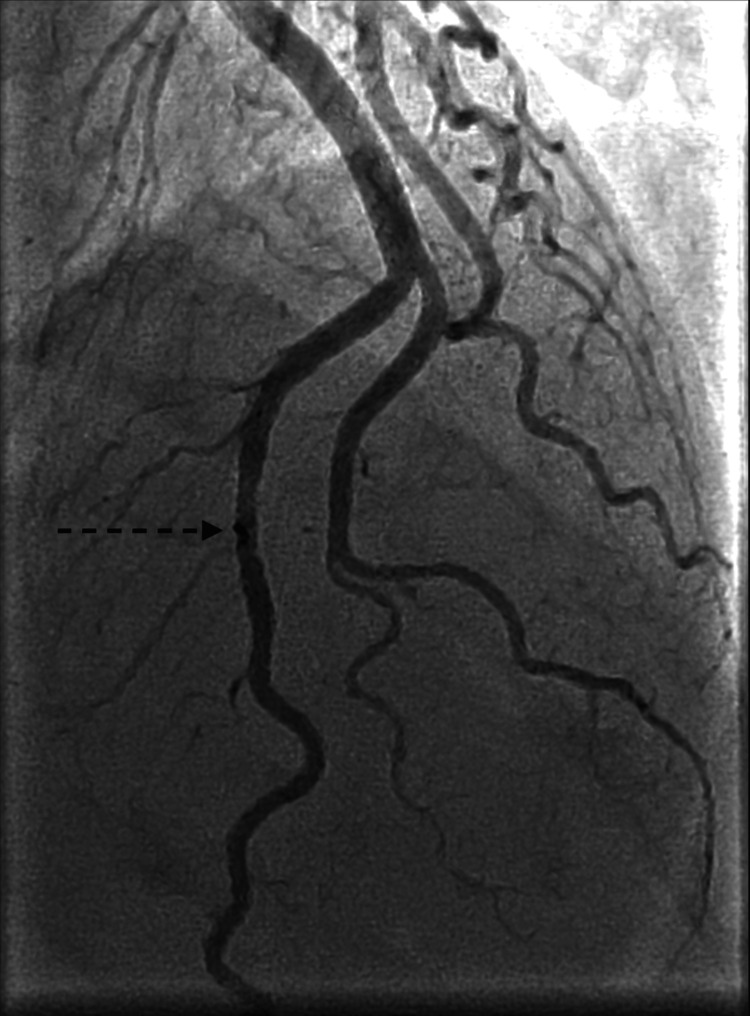
Type 3 spontaneous coronary artery dissection of the left anterior descending artery.

**Figure 2:**
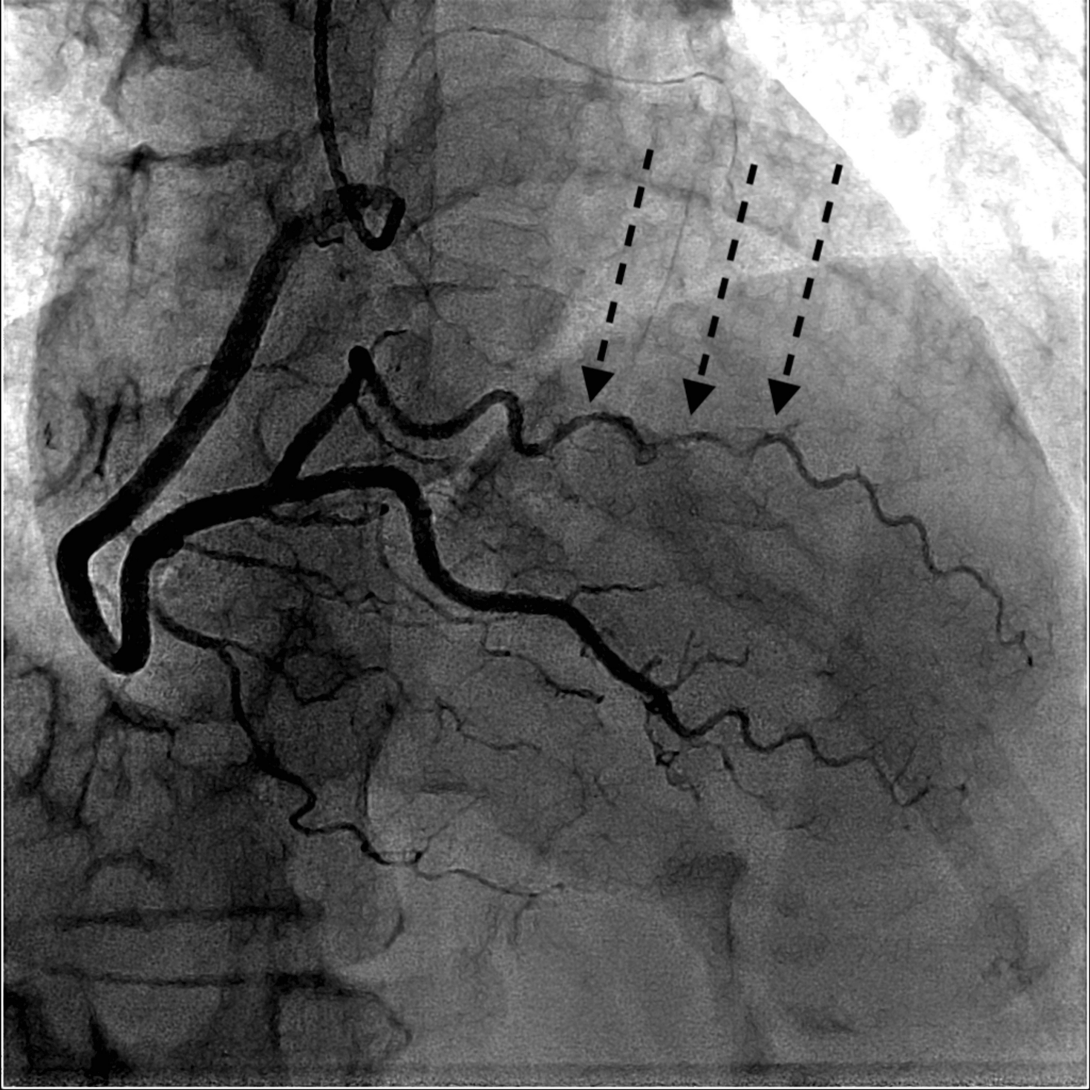
Type 2 spontaneous coronary artery dissection of the posterolateral branch of the right coronary artery.

Cardiac catheterization demonstrated a type 3 SCAD of the mid-left anterior descending artery and a type 2 SCAD of the mid-posterior left ventricular artery (Figures 1-2). There was no angiographic evidence of obstructive atherosclerotic coronary artery disease, and left ventricular end-diastolic pressure was normal.

**Figure 3:**
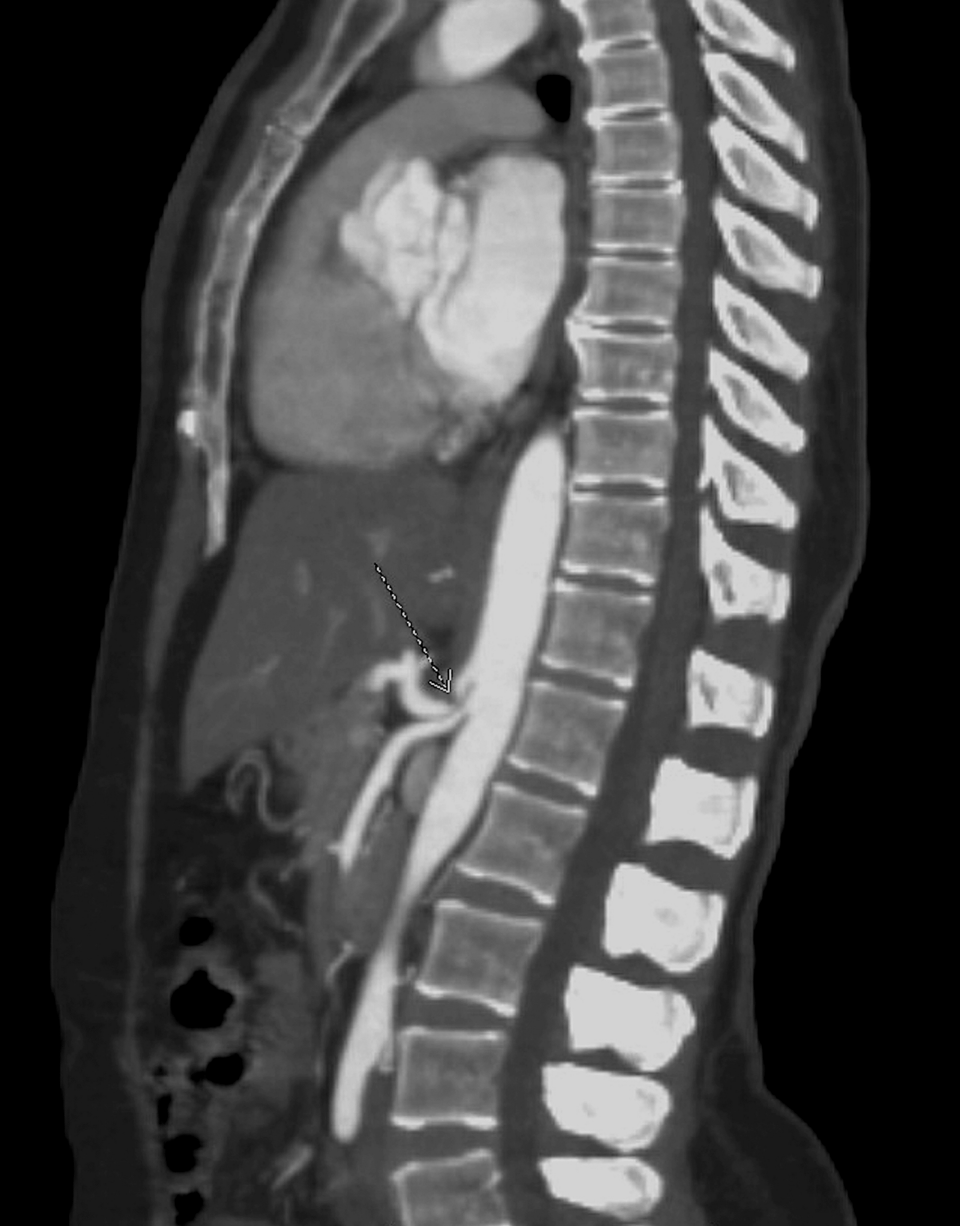
Abdominal and pelvic computed tomography angiography with contrast. The arrowhead indicates the hooked morphology of the celiac trunk, which is compatible with median arcuate ligament syndrome.

The electrocardiogram demonstrated a regular rate of 90 beats per minute and no evidence of ST elevations. The chest X-ray showed a regular cardiac silhouette, clear lungs, no edema, consolidations, or effusions, with mild bilateral lower lung atelectasis. An echocardiogram showed an ejection fraction of 55-60% with trace regurgitation of the mitral, pulmonary, and tricuspid valves. Additionally, it showed evidence that the middle inferior lateral wall was mildly hypokinetic. A computed tomography scan of the neck showed no evidence of fibromuscular dysplasia. It was significant for calcified plaque and moderate proximal left vertebral artery stenosis. A chest CT scan revealed no evidence of pulmonary embolism (PE). A CT scan of the abdomen and pelvis was significant for the celiac trunk's hooked morphology, which is compatible with ligament syndrome (Figure 3). The scan of the abdomen and pelvis showed no evidence of atherosclerotic disease, aneurysms, or dissections.

The only home medication the patient takes is escitalopram 20 mg to control her perimenopausal flashes. The patient is a nonsmoker who drinks alcohol, 1-2 drinks per week, and denies recreational drug use.

Based on the presentation of SCAD with no associated risk factors except mild hypercholesterolemia, we assume that the patient has FMD, considering the positive CT angiography finding of a hooked celiac trunk demonstrating vascular involvement as seen in patients with FMD. Median arcuate ligament syndrome (MALS) was also considered due to the hooked celiac trunk, a characteristic of MALS. However, the patient does not fit the clinical diagnostic criteria as she does not express abdominal pain, nausea, or vomiting as commonly seen in MALS.

Upon admission to the hospital, the patient was started on aspirin 81 mg, candesartan 8 mg once daily, metoprolol 25 mg daily, and rosuvastatin 40 mg once daily. She was placed on Lovenox (1 mg/kg) and did not have any additional episodes of chest pain during admission. The patient was followed up for one month after the SCAD event. She recovered and reported no new events.

## Discussion

SCAD is multifactorial, with various theories regarding its etiology. One explanation for its prevalence in females is that estrogen and progesterone may play a role in the connective tissue of the heart's vasculature, increasing the risk of dissection [2]. Another theory involves FMD, a systemic non-inflammatory vascular disease not associated with atherosclerosis, mainly affecting the renal and coronary arteries and causing stenosis, dissections, or aneurysms. In females younger than 50 years old, SCAD accounts for about 25% of acute coronary syndrome (ACS) cases [3]. In patients with SCAD and no risk factors, FMD is a potential underlying cause, and further workup, including digital subtraction angiography of commonly affected arteries like the renal and carotid, is warranted to confirm the diagnosis.

In 1958, McCormack LJ et al. first introduced FMD after studying three patients with arterial hypertension and renal artery stenosis. In 1964, Palubinskas AJ and Ripley HR described FMD as a pathology affecting the arterial system beyond the renal arteries in a case report detailing the involvement of FMD in the celiac artery in a 36-year-old female [4]. FMD is characterized by non-atherosclerotic, segmental fibroplasia of the intima, media, or adventitia, affecting the wall of small and medium-sized vessels from any artery, causing stenosis, aneurysms, or dissection [5-6]. FMD is classically diagnosed through the gold standard, invasive digital subtraction angiography, which illustrates the "string of beads" appearance of the renal artery or the appearance of stenosis, but other manifestations of FMD include vascular abnormalities as those seen in median arcuate ligament syndrome. Pathology is essential in diagnosing FMD; McCormack LJ et al. first described the pathological classification of renal artery FMD, detailing the involvement of fibroplasia at the levels of the intima, media, or adventitia [6]. Research shows that in patients with SCAD who are further evaluated for FMD with renal imaging, 86% of patients are found to have underlying FMD [7].

In the case presented, the patient was admitted for ACS, and she was found to have SCAD. In patients presenting with ACS and mild cardiovascular risk factors, SCAD should be on the list of differentials. The coronary catheterization procedure confirmed the presence of SCAD; however, a further workup for FMD is warranted given her lack of atherosclerotic risk factors, prompting CT angiography of the neck, chest, and abdomen/pelvis. The angiography was remarkable and positive for a hooked celiac trunk, as commonly seen in MALS. Although the diagnosis of MALS cannot be entirely ruled out, the patient did not have the abdominal pain, nausea, or vomiting commonly seen in individuals with MALS. It is highly possible that the positive angiography finding of the hooked celiac trunk represents a less common manifestation of FMD. One limitation to this proposed clinical diagnosis of FMD is that the patient is otherwise asymptomatic, and no other findings of vascular involvement were identified. There was no discussion about whether the patient had SCAD. However, the possibility of FMD was considered based on her lack of severe cardiovascular risk factors, female prevalence of the disease, and involvement of the hooked celiac trunk visualized on angiography. Another limitation is that no pathology was obtained from the patient to visualize and confirm the presence of fibroplasia. The patient was followed for only one month following the event, another limitation as the patient should have been followed up for at least 10 years as FMD would likely begin to develop associated symptoms such as hypertension or stroke. Research has found that FMD increases the risk of SCAD five-fold, and that in patients who have had a previous SCAD, 10% experience a recurrence [8].

The exact etiology of FMD is not known, and there is no current treatment to resolve or prevent the disease; however, there is value in educating patients on lifestyle modifications to decrease the risk factors for other cardiovascular conditions. In a retrospective cohort study from 2019, screening for FMD in SCAD patients was conducted with brain-to-pelvis imaging at the clinicians' discretion; 9 out of 90 SCAD patients had FMD [8]. Although there are no currently established recommendations, patients with SCAD should be evaluated for FMD, and those with evidence of FMD should be monitored regularly for elevated blood pressure and placed on necessary antihypertensives to prevent damage to the heart, kidneys, and brain.

## Conclusions

SCAD is a common cause of acute chest pain in females without significant comorbidities and can be identified using cardiac catheterization. Although the cause of SCAD appears to be multifactorial in nature, FMD is a known risk factor for SCAD, and therefore, it is important to consider FMD as a differential diagnosis in patients with acute coronary syndrome and the absence of cardiovascular risk factors. Due to the concomitant relationship between SCAD and FMD, it must be ruled out. Diagnosing FMD is challenging, and there is no known treatment for the disease. However, there is value in enhancing awareness and implementing screening for patients with FMD, especially those with SCAD, to improve clinical outcomes.
